# Quantitative Trait Locus Analysis: *Multiple Cross and Heterogeneous Stock Mapping*

**Published:** 2008

**Authors:** Robert Hitzemann, John K. Belknap, Shannon K. McWeeney

**Keywords:** Genetic theory of alcohol and other drug use, genetic factors, environmental factors, behavioral phenotype, behavioral trait, quantitative trait gene (QTG), inbred animal strains, recombinant inbred (RI) mouse strains, quantitative traits, quantitative trait locus (QTL) mapping, multiple cross mapping (MCM), heterogeneous stock (HS) mapping, microsatellite mapping, animal models

Until well into the 1990s, both preclinical and clinical research focused on finding “the” gene for human diseases, including alcoholism. This focus was reinforced by the emergence of technologies to either inactivate (i.e., knock out) a gene or add extra copies of an existing gene in a living organism, which clearly demonstrated that over- or underexpressing a single gene could have a profound effect on behavior. However, a small but vocal group of scientists, including many alcohol researchers, argued that behaviors, including alcohol-related behaviors, were complex traits and therefore no one gene likely would have a large effect. This view was consistent with a large body of genetic research conducted in plants and fruit flies (e.g., Paterson et al. 1988) indicating that, for example, even a presumably simple characteristic, such as the size of a tomato, was determined by several genes. However, it was difficult to convince the scientific community that, in terms of its genetic determination, behavior was similar to the size of a tomato. Only with the advent of new genetic tools did it become possible to prove that many different genes contribute to complex behavioral characteristics. These tools included the following (see Phillips 2002):

- Panels of recombinant inbred (RI) mouse strains. RI strains generally are generated by repeatedly inbreeding brother–sister pairs from the second-generation (F_2_) off-spring of two genetically distinct parent inbred strains. Each F_2_ animal has a slightly different combination of the parental genes. By repeated inbreeding of brother– sister pairs, researchers can generate numerous distinct inbred animal strains.
- Quantitative trait locus (QTL) mapping. Quantitative traits are characteristics such as height or sensitivity to alcohol that differ in the extent to which an individual possesses that characteristic. The variation in these traits is determined by both genetic and environmental factors. As noted above, the genetic contribution typically involves multiple genes, and each of these genes may exist in several variants (i.e., alleles). QTL analysis allows one to map, with some precision, the genomic position of these alleles.

For many researchers in the alcohol field, the breakthrough with respect to the genetic determination of alcohol-related behaviors occurred when Plomin and colleagues (1991) made the seminal observation that a specific panel of RI mice (i.e., the BXD panel) could be used to identify the physical location of (i.e., to map) QTLs for behavioral phenotypes. Because the phenotypes of the different strains in this panel had been determined for many alcohol-related traits, researchers could readily apply the strategy of RI–QTL mapping (Gora-Maslak et al. 1991). Although investigators recognized early on that this panel was not extensive enough to answer all questions, the emerging data illustrated the rich genetic complexity of alcohol-related phenotypes (Belknap 1992; Plomin and McClearn 1993).

The next advance came with the development of microsatellite maps (Dietrich et al. 1992, 1996). Microsatellites are short pieces of DNA characterized by the repetition of short (i.e., two to four nucleotide) sequences.^1^ The number of repetitions of some microsatellites differs among individuals or inbred strains and therefore can be used as a marker, allowing researchers to track how specific microsatellite sequences are inherited. Researchers have mapped the locations, of thousands of such microsatellites in the mouse as well as human genome. Tracking microsatellite markers at specific known sites in the genome is useful because one can simultaneously track the gene variants linked to these markers. With these tools available, the first QTL study mapping a behavioral trait (i.e., activity in a novel environment) in F_2_ offspring of two genetically distinct inbred mouse strains was published by Flint and colleagues (1995). This study detected numerous QTLs that were significantly associated with the behavior under investigation (see Lander and Kruglyak [1995] for a discussion of how a QTL is determined to be significant). Subsequently, there was an explosion of behavioral QTL mapping studies, including studies that focused on alcohol-related traits. In a summary of the behavioral mapping data in mice and rats, Flint (2003) reported that hundreds of QTLs had been detected and that, as expected, most of these had very small effects (i.e., accounted for less than 5 percent of the phenotypic variance). Although there has been no detailed summary of behavioral QTL mapping data since 2003, it is reasonable to assume that the number of QTLs detected just in animal models has increased by an order of magnitude.

Of course the easiest, most convenient strategy to map QTLs in mice would be to cross animals from two inbred strains that differ in the behavior under investigation (e.g., sensitivity to alcohol) and then study the offspring to identify relevant QTLs and eventually determine which gene located in the vicinity of the QTL actually is responsible for the observed effect. The main problem with mapping QTLs in such simple intercrosses is that the DNA region, in which the QTL most likely is located (i.e., the 95 percent confidence interval [CI]^2^ of the QTL), frequently is very large and may, in some cases, include an entire chromosome. Darvasi and Soller (1995) provided a simple equation^3^ to calculate the 95 percent CI. Based on this equation, if researchers used 600 F_2_ animals to map a QTL with an effect size of 5 percent, the DNA region that would contain the QTL with 95 percent certainty would encompass 25 centiMorgan (cM) or, for most chromosomes, between 35 and 50 million nucleotides—a region that typically contains hundreds of genes. To reduce this interval to a size that can be analyzed more easily (i.e., to about 1 cM), one would have to study 15,000 animals, which obviously is not feasible. It therefore seems safe to say that the issue of reducing the QTL interval (given the generally modest effect size of most behavioral QTLs) has been the biggest impediment in moving from identifying QTLs to identifying the actual quantitative trait gene(s) (QTGs) and eventually even the relevant nucleotides in those genes (i.e., the quantitative trait nucleotide[s] [QTNs]). Accordingly, relatively few QTGs have been identified unambiguously that contribute to behavioral phenotypes (e.g., Yalcin et al. 2004), and only one of these—a gene called *Mpdz*—is associated with an alcohol-related trait (i.e., acute alcohol withdrawal) (Fehr et al. 2002; Shirley et al. 2004).

In recent years, however, several strategies have emerged that may help reduce the QTL interval and thereby facilitate the identification of QTGs. This article briefly describes two approaches—multiple cross and heterogeneous stock mapping. Additional approaches are described in the following article by Denmark and colleagues (pp. 266–269).

## Multiple Cross Mapping

The concept of combining (i.e., integrating) data obtained from intercrosses of several inbred strains (i.e., multiple crosses) is being used widely to improve QTL characterization for traits of agricultural value (see, for example, Christiansen et al. 2006; Khatkar et al. 2004). The application of this approach, which has been termed multiple cross mapping (MCM), to traits of physiological and behavioral interest also is becoming more frequent (e.g., Hitzemann et al. 2000, 2002, 2003; Jagodic and Olsson 2006; Li et al. 2005; Malmanger et al. 2006; Park et al. 2003; Wergedal et al. 2007; Wittenburg et al. 2005). Our interest in MCM was triggered by the observation that QTL data generated by three different mouse F_2_ intercrosses in three different laboratories^4^ apparently all detected the same QTL on a part of mouse chromosome 1 that was associated with open-field activity (Flint et al. 1995; Gershenfeld et al. 1997; Koyner et al. 2000); however, the QTL was not detected in a cross of two other mouse strains^5^ (Hitzemann et al. 2000). Hitzemann and colleagues (2000) proposed that the information obtained with multiple crosses could be used to develop an empirical algorithm for sorting microsatellite markers in order to detect chromosomal regions with the highest probability of containing QTLs.

The principle underlying this theory was that since the inbred mouse strains used actually are closely related, the data described above suggests that there must be a region or regions on chromosome 1 where three strains (i.e., DBA/2J, BALB/cJ and A/J strains) are identical and different from the fourth strain (i.e., C57BL/6J strain). It is perhaps easiest to visualize this in binary terms, where 0 and 1 represent different nucleotides; in a region of interest, the three similar strains could have the structure “0100011100” while the C57BL/6J strain would have the structure “1011100011.” These different patterns are termed differences in haplotype structure. Accordingly, the three strains carry one unit of haplotype structure and the C57BL/6J strain carries a different unit. The haplotype difference could involve a single nucleotide polymorphism (SNP) or, as in the example above, multiple SNPs. Knowing the regions where the strains are similar and where they differ enhances QTL analyses because it provides additional information and thus greater statistical power. Even more details of this haplotype structure became available when researchers developed dense maps that showed the location of SNPs in multiple mouse strains (e.g., Wade et al. 2002). These maps confirmed that some regions of the genome contain very few SNPs, whereas others contain many SNPs. A QTL was presumed to have a greater likelihood of being associated with the SNP-dense region than with the SNP-poor region where there is very little genetic variation.

When conducting MCM analyses, researchers often use “crosses of convenience”—that is, they draw on data obtained in studies that they and other groups have conducted with the strains they were using to address specific research questions. One problem associated with this approach, however, is that often there are missing data. For example, consider the data that originally led to our development of MCM. The three studies on which the analysis was based involved four different inbred mouse strains, but only three different crosses of these animals were analyzed; data for the remaining possible crosses were not available. Without this information, however, the true haplotype structure of the QTL cannot be determined. To address this issue, we created a balanced panel of crosses from four inbred strains in which every strain was crossed with every other strain and have used this panel to map QTLs for open-field activity and alcohol-induced locomotion (Hitzemann et al. 2003; Malmanger et al. 2006). With this approach, the MCM algorithm markedly reduced the QTL CIs and correctly predicted QTL position and haplotype structure as determined by heterogeneous stock (HS) mapping, which is described in the following section.

## Heterogeneous Stock Mapping

The problem associated with conducting de novo MCM rather than using a convenience sample of already available, but incomplete, crosses is that it requires a lot of work and many animals. Assuming that, as described above, at least about 600 animals are needed to identify a QTL using an intercross of two inbred strains, then the genetic makeup (i.e., genotype) and relevant behavioral and physical characteristics (i.e., the phenotype) of 2,400 animals would have to be determined to obtain a balanced panel for four inbred strains. Although genotyping has become much easier with the availability of high-throughput devices to map SNPs, the overall effort is still considerable and costly. These considerations have prompted the emergence of HS mapping as an alternative strategy that is precise and provides good information on haplotype structure.

In heterogeneous populations, all individuals have diverse genetic backgrounds. For example, one commonly used heterogeneous mouse stock was generated by interbreeding animals from eight genetically diverse inbred strains (Phillips 2002). HS mapping was first described by Talbot and colleagues (1999) who used it to identify QTLs associated with the phenotype of open-field activity. The investigators were able to map numerous QTLs with high precision. However, the analyses did not detect QTLs associated with this phenotype that previously had been mapped in an F_2_ intercross population. Mott and colleagues (2000) provided a solution to this problem by developing a mapping algorithm termed HAPPY, which was designed to map QTLs in any HS population derived from known inbred strains without requiring further pedigree information.^6^ The HAPPY algorithm found the previously detected QTLs in the HS mapping and also determined that the QTLs had the expected haplotype structure. Knowing the QTL signature is of considerable value when integrating QTL, gene expression, and gene sequence data.

**Figure 15:**
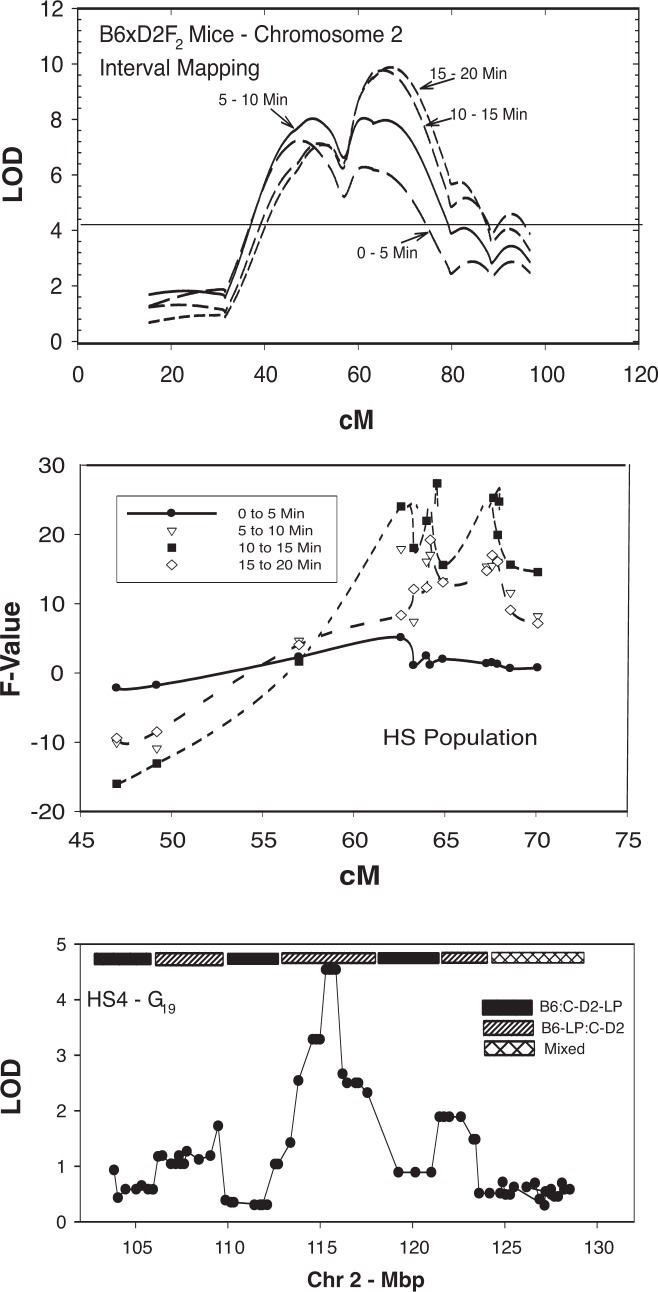
Three strategies for mapping a quantitative trait locus (QTL) on mouse chromosome 2 that is associated with acute ethanol locomotor response. The characteristic (i.e., phenotype) tested is the difference in activity between the administration of saline and the administration of 1.5 g/kg ethanol, measured in 5-minute intervals between 0 and 20 minutes after the injection. The top panel illustrates the result of a QTL mapping analysis in a C57BL/6J × DBA/2J F_2_ intercross (*N* = 600) (Demarest et al. 1999). The second panel illustrates mapping of the same phenotype in heterogeneous stock [HS-NPT] mice (*N* = 500) at generation 32 (Demarest et al. 2001). Data were analyzed in a marker-by-marker design; all markers were microsatellites and were classified as C57BL/6J–like or different. A positive F value indicates that a non-C57 allele is associated with an increased ethanol response. The HS analysis detected several QTLs that were not found in the F2 intercross analysis. The bottom panel shows the results of mapping the same phenotype using heterogeneous stock [HS4] animals (*N* = 575) at generation 19 and using a panel of closely spaced SNPs as markers (Malmanger et al. 2006). The bar at the top shows the haplotype structure across the region of interest. NOTE: The LOD (logarithm [base 10] of odds) is a measure of the degree of linkage between a given DNA region or gene and a specific trait.

There can be differences between the results achieved with HS mapping and those achieved with mapping in F_2_ intercross populations (see figure 15). For example, when analyses of a QTL on chromosome 2 that is associated with alcohol-induced locomotor response were conducted using F_2_ animals obtained by crossing C57BL/6J and DBA/2J mice, the resulting QTL interval was very broad (Demarest et al. 1999). Moreover, the investigators determined that those QTL alleles that the animals had inherited from the C57B6/6J mice were associated with a decreased response to alcohol. The same researchers then attempted to map the QTLs related to the ethanol response phenotype in an HS that was formed by crossing eight inbred mouse strains, including C57BL/6J and DBA/2J animals (Demarest et al. 2001). The analysis relied on microsatellite genotyping and simply classified alleles as either being similar to those found in C57BL/6J or being different from C57BL/6J alleles. This analysis detected multiple QTLs in the region of interest; furthermore, the C57BL/6J alleles were associated with both increased and decreased ethanol response. These findings suggest that the HR mapping approach is more sensitive than the F_2_ intercross approach and generates a greater variety of QTLs because none of the data suggest that these multiple QTLs also were present in the F_2_ intercross (although they also would have been invisible to the type of analysis used by Demarest and colleagues [1999]). Finally, Malmanger and colleagues (2006) performed QTL mapping for the ethanol response phenotype in an HS population generated by crossing four inbred strains (i.e., C57BL/6J, DBA/2J, LP/J, and BALB/cJ mice).^7^ This approach also detected a QTL peak in the region of interest that spanned a region of 1 to 2 million nucleotides. Moreover, the investigators determined the haplotype structure of the QTL and noted that the B6 allele was associated with decreased ethanol response. The integration of these data (i.e., position and haplotype of the QTL) with gene expression databases suggests a strong candidate QTG called *Scgn5* (also known as *7B2* and *Sgne1*), which encodes a protein called secretogranin 5 (Malmanger et al. 2006).

Currently, four mouse HS populations are available to investigators. One of these, the HS/Ibg, which was formed by crossing eight inbred laboratory mouse strains,^8^ is available through the Institute for Behavioral Genetics. The other three populations are maintained by the first author and include the HS4 population described in the previous paragraph, the HS– NPT population (see Valdar et al. 2006), and the HS–CC population (an eight-way cross that contains three mouse strains derived from the wild). These HS populations are freely available.

## Conclusion

QTL mapping has become an important aspect of efforts to determine the genetic basis of complex behaviors, such as alcohol-drinking behaviors. With new approaches to gene mapping, such as multicross mapping and HS mapping, which improve the accuracy with which QTLs can be located on the chromosomes, the identification of additional candidate QTGs likely is only a matter of time.

## Footnotes

1 Nucleotides are the building blocks of DNA. There are four different nucleotides called adenosine (A), cytosine (C), guanosine (G), and thymidine (T). Microsatellites are characterized by the repetition of two-to-four nucleotide pattens, such as CACACACA.

2 As with other parameters, the location of a QTL cannot be determined exactly based on measurements in just a few animals. Instead, a CI is calculated using statistical models that gives an estimated range of values which likely includes the unknown parameter. A 95 percent CI means that the likelihood of the estimated range including the actual value is 95 percent. The CI is calculated from a given set of sample data, and the more data are available, the more accurate the estimate will be and the smaller the range of values for the CI will be. A very wide CI indicates that more data should be collected before the parameter can be determined accurately.

3 According to this equation, CI = 1,500/Nd^2^, where *d* = the standardized gene effect; the constant 1,500 was determined empirically from computer simulations and is not related to genome size.

4 The studies involved crosses of C57BL/6J mice with BALB/cJ mice (Flint et al. 1995), A/J mice (Gershenfeld et al. 1997), and DBA/2J mice (Koyner et al. 2000).

5 This intercross involved BALB/cJ mice and LP/J mice (Hitzemann et al. 2000).

6 With this algorithm, the analysis basically occurs in two steps: (1) the ancestral haplotype is reconstructed using dynamic programming and then (2) QTLs are analyzed using linear regression.

7 The mapping was done at generation 19, which represents an approximately 10-fold expansion of the genetic map because with each generation additional recombinations are added that allow for finer mapping. In addition, the researchers used a dense SNP panel.

8 Two of these strains are no longer available.
